# Electrodermal Activity Biofeedback Alters Evolving Functional Brain Networks in People With Epilepsy, but in a Non-specific Manner

**DOI:** 10.3389/fnins.2022.828283

**Published:** 2022-03-03

**Authors:** Sophia Schach, Thorsten Rings, Madeleine Bregulla, Juri-Alexander Witt, Timo Bröhl, Rainer Surges, Randi von Wrede, Klaus Lehnertz, Christoph Helmstaedter

**Affiliations:** ^1^Department of Epileptology, University Hospital Bonn, Bonn, Germany; ^2^Helmholtz Institute for Radiation and Nuclear Physics, University of Bonn, Bonn, Germany; ^3^Interdisciplinary Center for Complex Systems, University of Bonn, Bonn, Germany

**Keywords:** electrodermal activity, biofeedback, epilepsy, evolving functional brain network, network characteristics, EEG

## Abstract

There is evidence that biofeedback of electrodermal activity (EDA) can reduce seizure frequency in people with epilepsy. Prior studies have linked EDA biofeedback to a diffuse brain activation as a potential functional mechanism. Here, we investigated whether short-term EDA biofeedback alters EEG-derived large-scale functional brain networks in people with epilepsy. In this prospective controlled trial, thirty participants were quasi-randomly assigned to one of three biofeedback conditions (arousal, sham, or relaxation) and performed a single, 30-min biofeedback training while undergoing continuous EEG recordings. Based on the EEG, we derived evolving functional brain networks and examined their topological, robustness, and stability properties over time. Potential effects on attentional-executive functions and mood were monitored *via* a neuropsychological assessment and subjective self-ratings. Participants assigned to the relaxation group seemed to be most successful in meeting the task requirements for this specific control condition (i.e., decreasing EDA). Participants in the sham group were more successful in increasing EDA than participants in the arousal group. However, only the arousal biofeedback training was associated with a prolonged robustness-enhancing effect on networks. Effects on other network properties were mostly unspecific for the different groups. None of the biofeedback conditions affected attentional-executive functions or subjective behavioral measures. Our results suggest that global characteristics of evolving functional brain networks are modified by EDA biofeedback. Some alterations persisted after the single training session; however, the effects were largely unspecific across the different biofeedback protocols. Further research should address changes of local network characteristics and whether multiple training sessions will result in more specific network modifications.

## Introduction

Epilepsy is a chronic neurological disorder characterized by either recurrent seizures, one unprovoked (or reflex) seizure and an increased probability for the occurrence of further seizures, or diagnosis of an epilepsy syndrome (Fisher et al., 2014). In about two-thirds of people with epilepsy, seizures can be well controlled with antiseizure medication (ASM), while one-third continue to have seizures despite appropriate drug treatment (Kwan and Brodie, 2000). For the latter group, several invasive, semi-invasive, and non-invasive treatments (e.g., epilepsy surgery, deep brain stimulation, responsive neurostimulation, invasive, or transcutaneous vagus nerve stimulation) can be successful non-pharmaceutical alternatives to achieve seizure freedom or at least a reduction in seizure frequency or severity (Carrette et al., 2015; Jobst and Cascino, 2015; Boon et al., 2018; von Wrede and Surges, 2021). In addition, complementary behavioral treatments for the management of seizures have gained interest since decades (Tang et al., 2014). Of these methods, electrodermal activity (EDA) biofeedback has recently been promoted as a potentially effective treatment (Nagai et al., 2019). EDA biofeedback is a non-invasive, cost-effective, and easy-to-apply therapeutic option without known risks. EDA is an indicator of changes in sympathetic autonomic nervous system activity that is closely linked to attention and emotion (Dawson et al., 2017). Using biofeedback, individuals learn to control physiological processes based on visual or auditory feedback. Nagai et al. (2004b) proposed an inverse relationship between peripheral sympathetic activity (reflected by EDA) and cortical excitability as assessed by the Contingent Negative Variation (CNV), which is an event-related slow cortical potential. The authors concluded that a biofeedback training aimed at increasing sympathetic arousal might lower cortical excitability and thus increase the seizure threshold. A subsequent study (Nagai et al., 2009) confirmed a sustained reduction of the CNV after several EDA arousal biofeedback sessions. Therefore, somewhat counter-intuitively, the aim of EDA biofeedback in people with epilepsy is to increase, rather than decrease, the level of peripheral sympathetic arousal (i.e., subjects are encouraged to increase skin conductivity).

The clinical effectiveness of this EDA biofeedback protocol has already been demonstrated in people with intractable epilepsy, with mean seizure reduction rates of around 50% as well as responder rates (subjects having ≥50% seizure reduction) of approximately 50% after 12 sessions of EDA biofeedback (Nagai et al., 2004c,^2018^; Micoulaud-Franchi et al., 2014; Kotwas et al., 2018). Furthermore, the aforementioned studies reported positive correlations between the increase in EDA over the biofeedback sessions and the extent of seizure reduction. However, a single case report (Scrimali et al., 2015) describes a significant reduction in seizure frequency after 2 years of EDA relaxation biofeedback (i.e., the inverse biofeedback protocol aimed at decreasing EDA).

As for the potential mechanisms and brain regions involved in EDA biofeedback, frontal deactivations during EDA biofeedback have already been demonstrated using functional MRI (Nagai et al., 2004a), indicating an involvement of frontally mediated attentional and executive functions in feedback learning and cognitive control. In addition, there is evidence of an involvement of the occipital cortices that process visual information as well as of cortical and subcortical areas that are associated with interoceptive awareness (e.g., anterior cingulate cortex, insula, amygdala) (Critchley et al., 2002). Such distributed network activations have also been reported for vagus nerve stimulation (VNS) (Rutecki, 1990; Ben-Menachem, 2002). For short-term transcutaneous auricular vagus nerve stimulation (taVNS), a topology-modifying, robustness- and stability-enhancing effect on functional brain networks has recently been demonstrated in people with epilepsy (von Wrede et al., 2021). Functional brain networks consist of nodes and edges, where nodes represent brain regions and edges represent functional interactions between pairs of nodes. Epilepsy is considered a network disease (Spencer, 2002), and different network characteristics can provide important indications regarding seizure precursors, seizure propagation, and seizure termination (Schindler et al., 2008; Bialonski and Lehnertz, 2013; Geier et al., 2015; Lehnertz et al., 2016; Kuhlmann et al., 2018; Rings et al., 2019; Fruengel et al., 2020; Zaveri et al., 2020).

In order to investigate whether there are effects of short-term EDA biofeedback on functional brain networks, we derived such networks from the EEG recorded before, during, and after an EDA biofeedback session. We applied a global analysis approach and calculated network characteristics representative of the networks’ topology, stability, and robustness. To examine the specificity of different biofeedback protocols, participants were allocated to three different biofeedback conditions (arousal, sham, relaxation). In addition, potential short-term effects of EDA biofeedback on frontally mediated attentional-executive functions as well as potential effects on self-rated mood and behavior were assessed before and after the biofeedback training.

## Materials and Methods

### Subjects

Thirty participants with proven diagnosis of epilepsy were recruited among inpatients of the Department of Epileptology of the University Hospital Bonn between November 2020 and April 2021. Participants were quasi-randomly assigned to one of three biofeedback groups (arousal biofeedback, sham biofeedback, relaxation biofeedback; *N* = 10 each). In order to ensure equal sample sizes in each group, we used a quasi-randomization procedure based on a predefined allocation sequence. Participants allocation to a group thus depended on the order of inclusion in the study. There were no differences between groups regarding demographic and clinical variables (see Table 1 for subject characteristics). All participants were required to stay in our long-term video EEG monitoring unit for diagnostic purposes. Exclusion criteria were the occurrence of seizures within 24 h before the biofeedback training, a diagnosis of genetic generalized epilepsy (GGE), previous brain surgery, invasive vagus nerve stimulation or deep brain stimulation, severe pre-existing cardiac conditions (e.g., myocardial infarction), language barrier, and mental disability. None of the participants had previous experience with EDA biofeedback.

**TABLE 1:** Demographic and clinical data per group.

	Arousal condition	Sham condition	Relaxation condition	Significance
*N*	10	10	10	
Sex (female)	70% (7)	50% (5)	60% (6)	χ*^2^* = 0.83, n.s.
Age	37.2 (13.2; 21–59)	35.8 (16.6; 20–63)	43.2 (17.6; 19–64)	*F* = 0.61, n.s.
Duration of epilepsy (yrs.)	7.1 (8.1; 1–27)	5.2 (4.6; 0.5–14)	11.6 (13.8; 1–32)	*F* = 1.16, n.s.
Number of ASM	1.7 (0.7; 1–3)	1.6 (0.7; 0–2)	1.7 (0.5; 1–2)	*F* = 0.09, n.s.
Total DDD	2.1 (1.5; 0.7–5)	2.2 (1.2; 0–3.5)	2.2 (0.7; 1.3–3.1)	*F* = 0.04, n.s.
EpiTrack score T1	34.2 (4.1; 29–41)	35.8 (2.5; 31–40)	32.6 (3.7; 26–39)	*F* = 2.12, n.s.
EpiTrack score T2	36 (4.5; 31–44)	37.7 (2.7; 33–42)	34 (4.1; 27–41)	*F* = 2.34, n.s.
NDDI-E	9 (2.5; 6–12)	11.4 (4.3; 8–20)	9 (2.9; 6–13)	*F* = 1.68, n.s.

*Data are given either in percentage (N in parentheses) or mean (standard deviation and range in parentheses). The last column shows results of testing for significant differences between groups (α = 0.05). Abbreviations: ASM, antiseizure medication; DDD, Defined Daily Dose; T1, pre-biofeedback; T2, post-biofeedback; NDDI-E, Neurological Disorders Depression Inventory for Epilepsy.*

The study was conducted with the full understanding and written consent of each participant, in accordance with a protocol approved by the ethics committee of the Medical Faculty of the University of Bonn (No. 294/20) and in accordance with the tenets of the Declaration of Helsinki.

Participants underwent a continuous EEG recording (phase 1: 60 min pre-biofeedback, phase 2: 30 min biofeedback training, phase 3: 60 min post-biofeedback) and a neuropsychological assessment preceding and following the EEG recording.

There were no prior changes in ASM from admission to our ward until completion of study participation. Adherence was ensured as medication is provided by the nursing staff in everyday clinical routine. Seizure provocation methods such as hyperventilation or sleep deprivation were not applied at least 24 h before the biofeedback training.

### Biofeedback Training and Electrodermal Recordings

**FIGURE 1:**
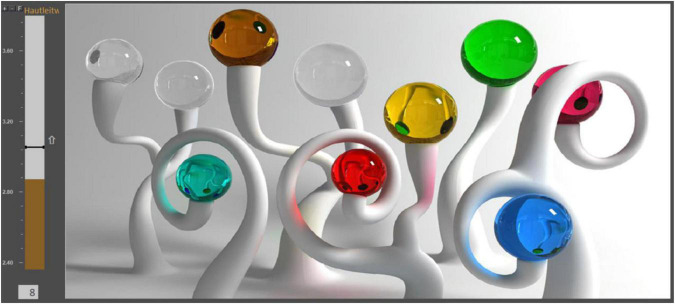
Feedback screen used in BioTrace+ (with kind permission of Mind Media BV, www.mindmedia.com).

We used the biofeedback system NeXus-4 (Mind Media BV, Herten, Netherlands) which was connected *via* Bluetooth to a laptop (Dell Latitude 3500) running the accompanying BioTrace+ software (Mind Media BV, Netherlands). EDA was recorded using the NeXus sensor with two Ag/AgCl electrodes imbedded into Velcro straps that were attached to the palmar surface of the index and middle finger of the non-dominant hand (according to standard methodology, see Boucsein et al., 2012). All participants were right-handed. Skin conductance data (in microsiemens, μS) were recorded with a sampling rate of 32 Hz. To evaluate specific effects of EDA arousal biofeedback, we implemented two control groups: (I) a sham condition, i.e., participants received the same instructions as participants in the arousal condition (namely, to increase EDA), but the feedback was not related to the actual EDA, and (II) an EDA relaxation biofeedback, i.e., the inverse protocol with the aim of decreasing EDA. Various visual feedback options are implemented in the BioTrace+ software. To keep the feedback identical for all three biofeedback conditions, we selected a neutral feedback that was neither directly associated with arousal nor with relaxation. The selected feedback screen (see Figure 1) showed an animation with ten initially transparent spheres that colored as the skin conductance value exceeded (for the arousal group) or fell below (for the relaxation group) an adaptive threshold described below. On the left side of the screen, a bar indicated the participant’s constantly changing EDA and the corresponding threshold that s/he had to exceed or fall below. The adaptive threshold was automatically updated taking into account the previous skin conductance value. It was calculated using the following formula provided by Mind Media BV: adaptive threshold = (previous threshold value * 127/128) + (1/128 * current EDA value). The mean of the first 128 EDA values served as the first threshold value. Given the sampling rate of 32 Hz, the threshold was based on the mean EDA of 4 s. The calculation is therefore comparable to the moving average technique from time series analysis. Participants typically went through multiple animation cycles as the animation started over if all ten spheres were colored. Participants in the sham condition were shown a pre-recorded half-hour arousal biofeedback session.

The biofeedback session consisted of an EDA baseline recording of 2 min in a neutral state (no feedback provided) and a subsequent 30-min biofeedback training. Participants were instructed to increase (or decrease) skin conductivity (e.g., by using attentional or cognitive strategies) while actively attending to the feedback screen. However, specific strategies were not provided. Participants were explicitly asked not to use breathing techniques to elicit a change in EDA. An investigator was present throughout the biofeedback session to monitor these factors. Phases of relevant increases or decreases in EDA that could clearly be attributed to artifacts (e.g., electrode readjustment) were excluded from analyses.

In order to obtain both a comprehensive picture of the recorded EDA and to describe the participant’s success in meeting the task requirements, we calculated the following three characteristics representing biofeedback performance:

(A) Change in skin conductance over the course of the biofeedback session was determined by subtracting the mean baseline skin conductance level (first 2 min without any feedback) from the mean skin conductance level of the last 2 min of the biofeedback training (see Nagai et al., 2004c). Furthermore, the percentage change from baseline to end of session was calculated.
(B) Individual performance was further assessed by calculating the percentage of time at which the participant’s skin conductance exceeded the adaptive threshold during the 30-min session.
(C) Finally, mean and standard deviation (SD) of the baseline EDA recording were calculated on a per-subject level. We defined a deviation of >2 SD from baseline level as a relevant change in EDA, and categorized all EDA raw values of the biofeedback session as “increase,” “decrease,” or “no change.” Frequencies of these three categories were calculated.

### Electroencephalographic Recordings, Data Pre-processing, and Network Characteristics

We used the same approach as in a previous study examining the effects of transcutaneous auricular vagus nerve stimulation (taVNS) on evolving functional brain networks in people with epilepsy (von Wrede et al., 2021). In the following, EEG recordings and network characteristics are described briefly (for a detailed description see von Wrede et al., 2021).

Electroencephalograms (EEG) were recorded from 19 electrode sites according to the 10–20 system with Cz serving as physical reference. All recordings were visually inspected for strong artifacts (e.g., subject movements, amplifier saturation), and such data were excluded from further analyses.

We used a sliding-window approach (Kuhnert et al., 2010; Dickten et al., 2016; Rings et al., 2019) to calculate a synchronization index (Mormann et al., 2000) from broadband EEG signals that serves as an indicator for the strength of functional interactions in the epileptic brain network (Kuhnert et al., 2013; Dickten et al., 2016). A synchronization index of 1 indicates fully phase-synchronized brain regions, while a synchronization index of 0 indicates no phase synchronization. In addition to the global synchronization level *R* (mean over all non-redundant pairwise synchronization indices), we assessed four relevant global characteristics for each network that we derived from the time-resolved synchronization analysis of the 2.5-h EEG recording prior to (phase 1), during [phase 2, biofeedback (BF)], and after the biofeedback training (phase 3). In order to remove possible transient effects, we neglected data from the first and last 15 min of the phases 1 and 3. This results in a similar duration of the three phases (30 min each) for the analysis.

Average shortest path length *L* and average clustering coefficient *C* were estimated to characterize the network’s global topology, while the network’s stability and robustness were assessed by estimating synchronizability *S* and assortativity *A*. Average shortest path length characterizes the network’s functional integration; the lower *L*, the more integrated is the network. Average clustering coefficient is a measure of the degree to which nodes in a network tend to cluster together. It characterizes the network’s functional segregation; the lower *C*, the more segregated is the network. Synchronizability *S* is a measure of the stability of the network’s synchronized state, and assesses the network’s propensity (or vulnerability) to get synchronized by an admissible input activation (Pecora and Carroll, 1998; Barahona and Pecora, 2002). The lower *S*, the more easily can the synchronized state be perturbed. Assortativity *A* assesses the tendency of edges to connect nodes with similar or equal properties. If edges preferentially connect nodes of similar (dissimilar) property, such networks are called assortative (disassortative). Disassortative networks are more vulnerable to perturbations and appear to be easier to synchronize than assortative networks.

### Neuropsychological Assessment and Subjective Measures of Wellbeing

Before and after the biofeedback training, all participants underwent a brief neuropsychological evaluation with the EpiTrack^®^ third edition (Helmstaedter, 2019). The EpiTrack^®^ is a screening tool for attentional-executive functions that consists of six subtests assessing response inhibition, visuo-motor speed, mental flexibility, visuo-motor planning, verbal fluency, and verbal working memory. The tests take a total of 15 min to complete. Based on the subtest results, an age-corrected total score is calculated (maximum score: 49 points after age-correction). A total score in the range of 29–31 points reflects mild impairment (>1 SD below the mean of the normative sample) and the cutoff score for severe impairment is ≤28 points (>2 SD below the mean of the normative sample). A significant intraindividual change in the EpiTrack^®^ scores between two assessments is reflected by a gain of ≥4 points or the loss of ≥3 points.

Finally, participants were asked to complete the Neurological Disorders Depression Inventory for Epilepsy [NDDI-E, cut-off score >15 (Gilliam et al., 2006)] and a modified version of the Adverse Events Profile (before and after EDA biofeedback) in order to assess self-perceived changes in the domains cognition (e.g., vigilance, psychomotor speed, ability to concentrate), behavior (e.g., mood, anxiety, restlessness), and physiological symptoms (e.g., dizziness, nervousness, headache). This was further supplemented by five items assessing calmness, the feeling of relaxation, nervousness, tension, and overstimulation (scored on a scale from 0 to 10). At the end of the biofeedback session, participants in the arousal and sham condition were asked to indicate whether they thought they were in the real or sham condition.

### Statistical Analyses

Differences in the biofeedback performance characteristics between biofeedback groups as well as intraindividual changes in EDA over the course of the biofeedback session were investigated using Kruskal–Wallis tests or Wilcoxon tests, respectively (*p* < 0.05).

Differences between network characteristics from the three phases (phase 1: pre-biofeedback; phase 2: biofeedback training; phase 3: post-biofeedback) were investigated on group level (arousal vs. sham, sham vs. relaxation, arousal vs. relaxation) as well as on a per-subject level using the Mann-Whitney *U*-test (phase 1 vs. phase 2, phase 1 vs. phase 3, and phase 2 vs. phase 3; *p* < 0.05). Relative changes of the network characteristics between the three phases were calculated as Δ = (M_l_ − M_k_)/M_k_, where M_k_ and M_l_ denote placeholders for the temporal means of the respective characteristics from phase k and phase l.

Mean changes in the EpiTrack score from pre- to post-biofeedback were analyzed using a repeated measures ANOVA with time (pre- vs. post-biofeedback) as within-subjects factor and group (arousal, sham, relaxation) as between-subjects factor.

## Results

### Biofeedback Performance

No difference in baseline EDA was observed between groups (Kruskal–Wallis test; *H* = 0.91, *p* = 0.64). When comparing EDA between baseline and end of the biofeedback session, EDA did not change significantly over the course of the biofeedback session in any of the three groups (all *p*-values > 0.05), and percentage change did not differ significantly between groups (*H* = 4.45, *p* = 0.11). Similarly, there was no difference between groups in the percentage of time at which the skin conductance exceeded the adaptive threshold (between 27.8 and 33.5% of time for the different biofeedback conditions, see Table 2; *H* = 0.98, *p* = 0.61). However, keeping in mind that the aim of the participants in the relaxation condition was different from that in the arousal and sham condition (participants in the relaxation condition were asked to fall below the threshold by keeping the EDA low instead of increasing it as in the arousal and sham condition), the overall success seems to be higher in the relaxation group because they met the goal of falling below the threshold in 71% (on average) of the time.

**TABLE 2:** Biofeedback performance characteristics.

	Arousal condition	Sham condition	Relaxation condition
Change in EDA (in μS) from baseline to end of session	−0.17 (0.93), Mdn = 0.05	0.31 (1.34), Mdn = 0.25	−0.63 (1.15), Mdn = −0.17
Percentage change in EDA from baseline to end of session	0.8 (41.2), Mdn = 3.7	26.3 (54.9), Mdn = 20.2	−18.9 (28.6), Mdn = −19.2
% time at which the EDA exceeded the threshold	27.8 (8.4), Mdn = 27	33.5 (10.9), Mdn = 32.4	28.8 (7.4), Mdn = 32.4
% time at which EDA was >2 SD above baseline level	31.3 (39.9), Mdn = 10.1	55.5 (39.5), Mdn = 60.4	22.7 (36.3), Mdn = 4.3
% time at which EDA was >2 SD below baseline level	49.6 (46.1), Mdn = 52.4	18 (26.5), Mdn = 1.6	44.1 (43.2), Mdn = 40.2

*Data are given in mean values (SD in parentheses), followed by the median (Mdn). Abbreviations: EDA, electrodermal activity.*

We observed no group difference in the amount of time at which the participant’s EDA during the biofeedback training was at least 2 SD above (*H* = 3.78, *p* = 0.15) or below (*H* = 2.78, *p* = 0.25) their mean baseline EDA (see Table 2). However, at a descriptive level, participants in the sham group kept their EDA above the individual baseline level a higher proportion of the time (Mdn = 60%) than participants in the arousal group (Mdn = 10%). As could be expected, the least amount of time above baseline level was observed in participants in the relaxation group (Mdn = 4%).

One participant in the sham group correctly assumed that s/he was assigned to the sham condition, while two participants in the arousal group thought that they received the sham training.

### Effects of Electrodermal Activity Biofeedback on Evolving Epileptic Brain Networks

**FIGURE 2:**
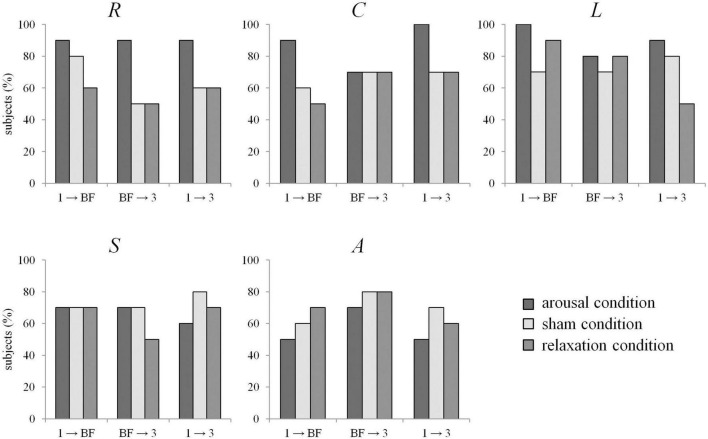
Percentage of subjects for which EDA biofeedback led to significant differences between network characteristics from phases 1 (pre-biofeedback), BF (biofeedback-training), and 3 (post-biofeedback), separate for the arousal condition (dark gray), sham condition (light gray), and relaxation condition (medium gray). *R*: global synchronization level, *C*: average clustering coefficient, *L*: average shortest path length, *S*: synchronizability, *A*: assortativity.

No significant differences in the network characteristics between the different groups were observed in a group-level analysis (Mann-Whitney *U*-tests, *p* > 0.05). Given the small sample size and considering that not every participant was equally successful in meeting the task requirements of the biofeedback training, we further analyzed the data on an individual level. Significant immediate (phase 1 → biofeedback) and enduring (phase 1 → phase 3) alterations of the global synchronization level *R* were seen in 9 out of 10 participants (90%) assigned to the arousal condition. In the sham and relaxation group, significant changes could be observed in a smaller amount of participants (50–80% across transitions, see Figure 2). Looking at the networks’ topology, we see that average clustering coefficient *C* was also more likely to be modified in the arousal group (immediate and enduring changes in 90–100%), compared to both other groups (50–70%). Similarly, for average shortest path length *L* it should be emphasized that significant immediate changes were seen in 100% of the participants in the arousal group, but with frequent immediate changes also shown in the sham and relaxation groups (70–90%). Synchronizability *S* and assortativity *A* were found to be significantly altered in a similar number of participants in each group (see Figure 2).

**FIGURE 3:**
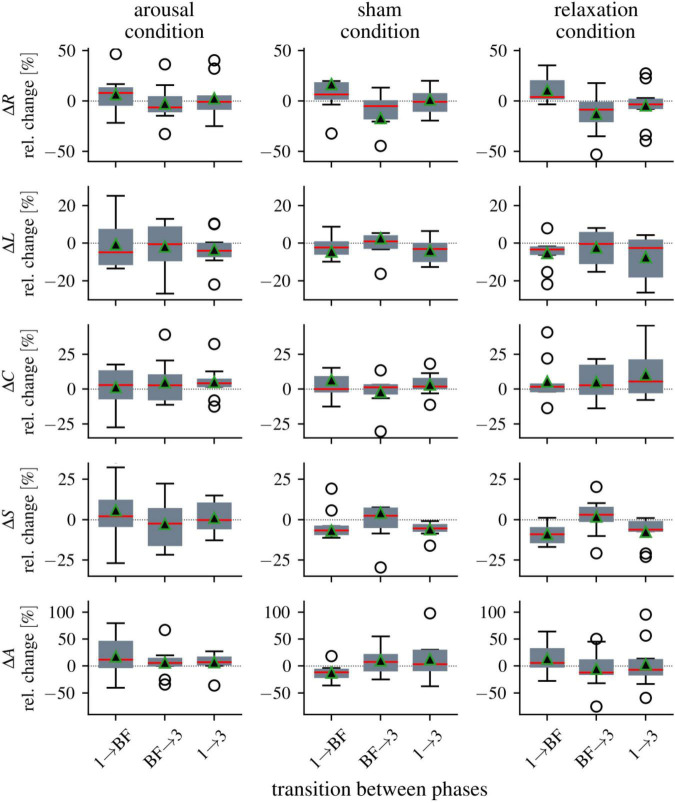
Distributions of alterations in global network characteristics between the three phases (1 = pre-biofeedback, BF = biofeedback-training, 3 = post-biofeedback), separate for the three biofeedback conditions. Boxplots of relative changes in network characteristics (global synchronization level *R*, average clustering coefficient *C*, average shortest path length *L*, synchronizability *S*, and assortativity *A*). Bottom and top of a box are the first and third quartiles. The red band and the black triangle are the median and the mean of the distribution, respectively. The ends of the whiskers represent the interquartile range of the data. Outliers are marked by an o-sign.

We next calculated the relative changes (median values) for the network characteristics between the three phases, which we present below separately for the three groups, i.e., all ten participants per group were included in the calculation of the median values (see Figure 3 and Table 3). For the global synchronization level *R*, we observed an increase from pre-biofeedback to biofeedback in all groups (phase 1 → biofeedback; arousal: 8%, sham: 7%, relax: 4%). Since *R* decreased after the training in each group (biofeedback → phase 3; arousal: −6%, sham: −5%, relax: −8%), pre- and post-biofeedback levels were comparable (relative changes phase 1 → phase 3: ≤3%).

**TABLE 3:** Synopsis of EDA biofeedback-induced immediate (phase 1 → biofeedback) and enduring (phase 1 → phase 3) modifications of global network characteristics in the different biofeedback conditions (*N* = 30).

		Arousal condition	Sham condition	Relaxation condition
Topology	Immediate effect	Segregation ↑ Integration ↓	Segregation ↔ Integration ↔	Segregation ↔ Integration ↓
	enduring effect	Segregation ↑ Integration ↓	Segregation ↔ Integration ↓	Segregation ↑ Integration ↔
Robustness	Immediate effect	↑↑	↓↓	↑
	enduring effect	↑↑	↔	↓
Stability	Immediate effect	↔	↓	↓
	enduring effect	↔	↓	↓

*↑↑ or ↓↓ = relative changes ≥ 10%; ↑ or ↓ = relative changes between 2 and 10%; ↔ = relative changes ≤ 2%.*

For average clustering coefficient *C*, we observed enduring increases in the arousal and relaxation groups (phase 1 → phase 3: 4 and 6%, respectively). All other relative changes of average clustering coefficient *C* between phases range between 0 and 3%. Average shortest path length *L* as another topological network characteristic decreased in all groups immediately and persistently (relative changes between phase 1 and biofeedback as well as between phase 1 and phase 3 range between −3 and −5%).

While synchronizability *S* decreased from pre-biofeedback to the biofeedback phase in the sham and relaxation groups (phase 1 → biofeedback: −7 and −9%, respectively), we observed a slight increase in the stability of the networks in the arousal group (2%). The decreases in the sham and relaxation groups were persistent (phase 1 → phase 3: −5 and −6%, respectively) but the post-biofeedback level in the arousal group was comparable to the pre-biofeedback level (phase 1 → phase 3: −0.2%).

Assortativity *A* increased with the biofeedback training in the arousal and relaxation groups (phase 1 → biofeedback: 12 and 6%, respectively), but decreased in the sham condition (−11%). The increased robustness of the networks in the arousal group was maintained (phase 1 → phase 3: 10%). In contrast, *A* decreased in the relaxation group after biofeedback (biofeedback → phase 3: −12%) resulting in a decrease from pre- to post-biofeedback (phase 1 → phase 3: −7%). No enduring changes could be observed in the sham group (phase 1 → phase 3: −1%).

Finally, we again calculated relative changes in the network characteristics between phases, but considered only data of those participants for whom we could identify significant differences in the network characteristics between the phases (see Figure 2). As expected, we observed more pronounced relative changes for these subsamples of EDA biofeedback “responders” compared to the above described median values for the total groups (i.e., all ten participants per group). Particularly noteworthy is the robustness (assortativity) of the networks for which we observed relative changes of more than 50% in the arousal group when networks transited to the biofeedback phase (phase 1 → biofeedback; arousal: 53%, sham: −19%, relax: 24%). The robustness was persistently increased (albeit to a lesser extent) in the arousal group (phase 1 → phase 3: 16%), but decreased in the relaxation group (phase 1 → phase 3: −16%). Since the directions of change of the different network characteristics between phases as well as the partially opposite effects for the different groups were consistent with the above descriptions for the total groups, we do not describe the subgroup results in further detail.

### Neuropsychological Evaluation and Subjective Measures

Before the biofeedback training, performance in the EpiTrack^®^ was impaired in 26.7% (8 out of 30) of participants (arousal = 3/10, sham = 1/10, relax = 4/10).

Executive functioning as measured by the EpiTrack^®^ improved from pre- to post-assessment (main effect *time*: *F* = 37.22, *p* < 0.001), but there was no interaction effect of time and group (*F* = 0.3, *p* = 0.74). Two participants in the relaxation condition and one participant in the sham condition showed a significant intraindividual improvement in the EpiTrack^®^ (≥4 points). None of the participants worsened significantly.

According to the NDDI-E cutoff score, two participants (both in the sham condition) showed depressive symptoms. No significant self-perceived changes were observed regarding the total scores in the cognitive, behavioral, and physiological domain of the modified Adverse Events Profile.

## Discussion

The primary objective of the current study was to investigate potential alterations of functional brain networks induced by a single, 30-min EDA biofeedback training session implementing three conditions (arousal, sham, and relaxation). The study was motivated by previous reports indicating a beneficial effect of EDA arousal biofeedback on seizure control (for a systematic review see Nagai et al., 2019). Furthermore, in a previous study (von Wrede et al., 2021) which investigated the effects of short-term transcutaneous auricular vagus nerve stimulation (taVNS) on functional brain networks, modifications of the networks’ topology as well as robustness- and stability-enhancing effects of taVNS were observed. In our current study, we applied the same analysis approach as in the study mentioned above to explore if and how EDA biofeedback may alter network characteristics.

Thirty participants with proven diagnosis of epilepsy were recruited and quasi-randomly assigned to one of the three biofeedback conditions. Participants in the arousal group presented with frequent changes of the global synchronization level and of the networks’ topological organization. In addition, we observed a prolonged robustness-enhancing effect on functional brain networks that was specific to the arousal group. Relative changes of other network characteristics, however, were mostly unspecific for the different groups. Neither biofeedback protocol resulted in differential short-term changes in attentional-executive functions or subjective behavioral measures.

### Electrodermal Activity Biofeedback Performance

For the following interpretation of the results, it is important to keep in mind that many participants failed to systematically control their EDA in the desired direction within one session. Sympathetic activity (as measured by EDA) did not change significantly in any of the groups between baseline and the end of the session. Contrary to expectations, on a descriptive level, the greatest percentage increase in EDA was observed for the sham condition. Similarly, the percentage time at which the participants maintained their EDA above baseline level (>2 SD) was highest for the sham condition. The term “sham” implies that this condition was meant to have no effects on the actual sympathetic activity. Our results, however, question this assumption. Participants in the arousal and sham condition received the same instructions (namely, to increase EDA), but in the sham condition the participants’ efforts to increase their EDA did not result in changes in the feedback screen. Although the following interpretation is speculative, this potentially frustrating experience in the sham group might have been a stronger stimulus than the learning process itself based on the feedback in the active biofeedback group. However, in our sample, only one participant recognized the sham group as such. As expected, EDA tended to decrease for the relaxation condition and the amount of time spent above baseline level was the lowest of all three groups. Since most people have a concept of relaxation in mind, the state of relaxation might be more accessible and easier to evoke than a conscious increase in sympathetic activity.

One might assume that the difficulties of the participants to successfully meet the task demands (i.e., increase or decrease EDA) are due to the fact that this skill has to be learned over several sessions, as was the case in the studies that revealed positive effects on seizure control (for a review see Nagai et al., 2019). However, Nagai et al. (2004b) demonstrated a 10-min arousal biofeedback training to specifically affect the CNV. In an fMRI study with 6 min of EDA arousal and relaxation biofeedback each, Nagai et al. (2004a), in agreement with our results, also reported a greater behavioral success of participants to decrease (i.e., relax) compared to increase their EDA, and found task-independent modulations of activity within the ventromedial prefrontal cortex and the orbitofrontal cortex. Regarding biofeedback performance, the authors stated that “this skill can be learned in principle after only few minutes of practice, although overall performance ability may vary markedly across subjects” (Nagai et al., 2004a). Biofeedback can be considered as a type of operant conditioning (Frank et al., 2010), since the control of physiological processes happens through feedback learning and positive reinforcement (e.g., continuation of the feedback animation). It is conceivable that learning biofeedback is generally impaired in people with epilepsy due to the underlying pathophysiology. It has already been shown that people with epilepsy exhibit deficits in conditioning paradigms, e.g., in classical eye-blink conditioning, which has been attributed to cerebellar atrophy in temporal lobe epilepsy (TLE) (Hermann et al., 2004), or in fear conditioning, for which structures of the medial temporal lobe (especially the amygdala) play a decisive role (LaBar et al., 1995; Weike et al., 2005). The amygdala is also crucially involved in electrodermal responses (Critchley, 2002; Dawson et al., 2017), and reduced electrodermal responses to emotional stimuli have been observed in participants with (autoimmune) TLE and in participants with psychogenic non-epileptic seizures (Holtmann et al., 2018; Kotwas et al., 2019). The fact that 70% of the participants in our sample were diagnosed with TLE (of whom nearly 60% had confirmed or suspected limbic encephalitis) should be taken into account when interpreting the difficulties in biofeedback performance. Interestingly, a recent study (Schönenberg et al., 2021) on attention-deficit/hyperactivity disorder pointed out the importance of participants’ expectations concerning neurofeedback therapy: following the induction of positive expectancies on the neurofeedback’s effectiveness (“placebo” condition), participants perceived their attentional performance as improved (compared to “no expectancy” and “nocebo” conditions). Subjective expectancy and believes should also be assessed when investigating EDA biofeedback.

### Biofeedback-Induced Network Modifications

Despite the unexpected biofeedback performance results, we were able to detect changes in network characteristics, some of which were group-specific. Individual-level analyses indicated that all but one participant in the arousal group showed a change in the global synchronization level *R* that persisted after the end of training. Furthermore, we observed opposite, group-specific effects for assortativity *A*, which point to a prolonged robustness-enhancing effect of arousal EDA biofeedback. The observation that the networks’ robustness first increased in the relaxation condition, but fell below the initial level after training, may indicate that the networks can be more easily synchronized in the long term due to a reduction in sympathetic activity. This would be in line with the theory of Nagai et al. (2004b) which proposes that reducing EDA might have a detrimental effect on seizure control because of the negative relationship between peripheral sympathetic activity and cortical excitability (see also Kotwas et al., 2015). Such considerations, however, remain at a highly speculative level and require further investigation. For synchronizability *S*, we observed an enduring decrease for the sham and relaxation conditions, indicating a higher resilience against perturbations. Surprisingly, there was hardly any change in synchronizability in the arousal group.

Considering these partially contradictory results, we hypothesize that one and the same action (increasing or decreasing sympathetic activity) can theoretically be either seizure-promoting or anticonvulsant at different times, depending on what level of arousal the patients are at a particular point of time. This hypothesis refers to the concept of recruitment and availability, i.e., when the brain is functionally occupied (e.g., engaged in mental activity), there is less capacity for epileptic activity (Speckmann, 1986; Helmstaedter, 2008). Overall, it might be crucial to train the flexibility of the sympathetic system in both directions, so that participants can develop explicit or implicit strategies to either increase or decrease their sympathetic activity depending on their current level of arousal. This might be particularly helpful for subjects who experience auras or prodromes as warning signs before the occurrence of a seizure, so that an impending seizure could potentially be prevented by using certain strategies. Wearables such as biosensor wristbands could help to monitor the electrodermal activity (Poh et al., 2010; Vieluf et al., 2020).

Recent studies investigated functional connectivity after repeated EDA arousal biofeedback sessions in people with epilepsy. Ferri et al. (2021) evaluated magnetoencephalography (MEG) resting-state functional networks and found a decrease of the WPLI-beta-low (weighted phase lag index) value in biofeedback responders (reduction of seizure frequency ≥ 50%). Although their sample size was very small (*N* = 6; 2 responders), their results, in agreement with our findings, support the idea that EDA biofeedback might modify functional brain networks since WPLI is comparable to the synchronization index *R* that we employed (Porz et al., 2014). In an fMRI study, Nagai et al. (2018) reported an increase in functional connectivity between right amygdala and the orbitofrontal cortex as well as the frontal pole in correlation with seizure reduction, while neuroimaging studies examining brain activity associated with EDA relaxation biofeedback suggest a crucial role of the anterior cingulate cortex, among several other areas (Critchley et al., 2001, 2002). Overall, studies to date thus do not yet provide a clear picture of brain mechanisms underlying EDA biofeedback (for a detailed description of brain mechanisms related to the generation and control of electrodermal responses see Critchley, 2002).

### Biofeedback-Induced Modifications of Cognition and Behavior

As EDA biofeedback seems to be associated with the activation of frontal brain structures (among others), it could have been expected that the training might lead to changes in performance in tests that are thought to assess frontally mediated attentional-executive functions. Taking into account that only a few participants showed impaired performance in the EpiTrack^®^ at baseline, neither relevant improvements nor deteriorations from pre- to post-biofeedback were evident at the individual level. Participants seemed to feel comfortable during the EDA biofeedback, and the training did not lead to feelings of tension or overstimulation (which would have been conceivable especially for the arousal and sham arousal conditions).

### Study Limitations

A main limitation to the interpretability of the described network modifications is that the EDA biofeedback training as performed here did not result in significant within- or between-group differences in EDA. The generalizability of our results is limited by the small sample size and the heterogeneity of the participants’ epilepsy-related variables (e.g., different etiologies, lateralization/localization of seizure onset zone). Taking into account the feedback of some participants, the training duration of 30 min might have been too long. Multiple shorter sessions during the day could be more appropriate. Since our data were collected in a clinical setting, it was not possible to extend the EEG recording solely for study purposes.

## Conclusion and Future Perspectives

In conclusion, our results suggest that EDA biofeedback alters global characteristics of evolving functional brain networks; however, the effects were largely unspecific for the different biofeedback conditions after a single training session. Some of the induced changes lasted at least an hour after the end of training. Further studies are needed to explore longer-term effects on functional brain networks after several training sessions that could help to uncover neuronal mechanisms of action of EDA biofeedback, especially since there are hints that the reduction of the seizure frequency might be sustained (Nagai and Trimble, 2014). Furthermore, it would be insightful to train different directions (increasing versus decreasing EDA) in an intraindividual study design to further explore the specificity of the different EDA biofeedback protocols.

In the current study, we focused exclusively on global network characteristics of large-scale functional brain networks, however, deeper insights into the networks’ dynamics related to EDA biofeedback may be gained using local analyses approaches on the scale of single nodes and edges based on centrality indices (Geier and Lehnertz, 2017; Bröhl and Lehnertz, 2019; Rings et al., 2021). This approach would allow to assess the importance of specific brain regions and their interactions within the larger evolving functional brain network.

## Data Availability Statement

The datasets presented in this article are not readily available because they contain information that could compromise the privacy of research participants. Requests to access the datasets should be directed to corresponding author SS, sophia.schach@ukbonn.de.

## Ethics Statement

The studies involving human participants were reviewed and approved by the Ethics committee of the Medical Faculty of the University of Bonn. The patients/participants provided their written informed consent to participate in this study.

## Author Contributions

All authors contributed to conception and design of the study, manuscript revision, read, and approved the submitted version. MB and SS performed the experiments. TR and SS analyzed the data and performed the statistical analysis, with valuable input of CH, KL, and J-AW. SS wrote the first draft of the manuscript.

## Conflict of Interest

The authors declare that the research was conducted in the absence of any commercial or financial relationships that could be construed as a potential conflict of interest.

## Publisher’s Note

All claims expressed in this article are solely those of the authors and do not necessarily represent those of their affiliated organizations, or those of the publisher, the editors and the reviewers. Any product that may be evaluated in this article, or claim that may be made by its manufacturer, is not guaranteed or endorsed by the publisher.
